# Endosymbiotic and Host Proteases in the Digestive Tract of the Invasive Snail *Pomacea canaliculata*: Diversity, Origin and Characterization

**DOI:** 10.1371/journal.pone.0066689

**Published:** 2013-06-20

**Authors:** Martín S. Godoy, Alfredo Castro-Vasquez, Israel A. Vega

**Affiliations:** 1 Instituto de Fisiología, Facultad de Ciencias Médicas, Universidad Nacional de Cuyo), Mendoza, Argentina; 2 Instituto de Histología y Embriología “Dr. Mario H. Burgos”, Consejo Nacional de Investigaciones Científicas y Técnicas, Mendoza, Argentina; 3 Instituto de Ciencias Básicas, Universidad Nacional de Cuyo, Mendoza, Argentina; University of Chicago, United States of America

## Abstract

Digestive proteases of the digestive tract of the apple snail *Pomacea canaliculata* were studied. Luminal protease activity was found in the crop, the style sac and the coiled gut and was significantly higher in the coiled gut. Several protease bands and their apparent molecular weights were identified in both tissue extracts and luminal contents by gel zymography: (1) a 125 kDa protease in salivary gland extracts and in the crop content; (2) a 30 kDa protease throughout all studied luminal contents and in extracts of the midgut gland and of the endosymbionts isolated from this gland; (3) two proteases of 145 and 198 kDa in the coiled gut content. All these proteases were inhibited by aprotinin, a serine-protease inhibitor, and showed maximum activity between 30°C and 35°C and pH between 8.5 and 9.5. Tissue L-alanine-N-aminopeptidase activity was determined in the wall of the crop, the style sac and the coiled gut and was significantly higher in the coiled gut. Our findings show that protein digestion in *P. canaliculata* is carried out through a battery of diverse proteases originated from the salivary glands and the endosymbionts lodged in the midgut gland and by proteases of uncertain origin that occur in the coiled gut lumen.

## Introduction

*Pomacea canaliculata* (Lamarck 1822) (Caenogastropoda, Ampullariidae) is a highly invasive apple snail original from Central and Northern Argentina, Southern Brazil and Uruguay, and that has spread to Southeast Asia, North America and Europe where it has become a plague for rice and other crops [1], [2], [3].

**Figure 1:**
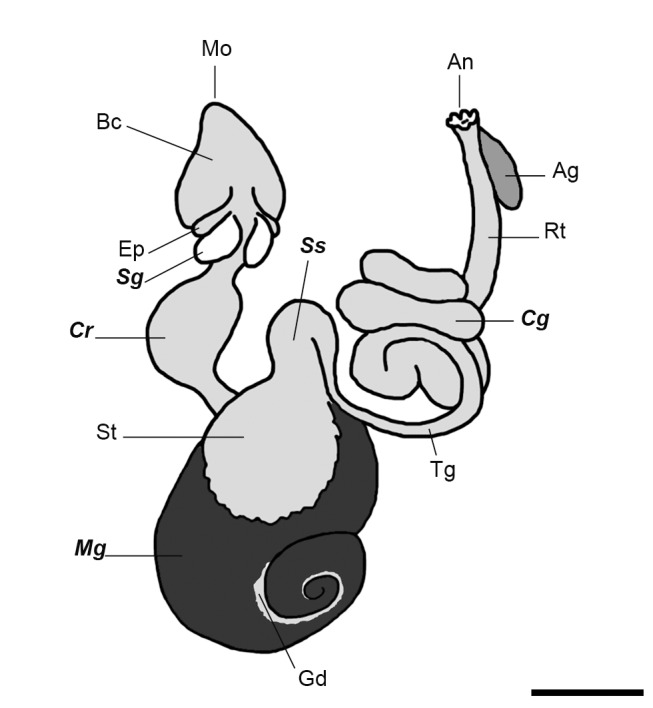
Schematic view of the digestive system of *Pomacea canaliculata*. In aboral order: mouth (Mo), buccal cavity (Bc), esophagic pouches (Ep), salivary glands (***Sg***), crop (***Cr***), stomach (St), midgut gland (***Mg***) with gonad (Gd), style sac (***Ss***), thin gut (Tg), coiled gut (***Cg***), rectum (Rt), anal gland (Ag) and anus (An). Bold italic letters indicate the sampled tissues and luminal contents. Scale bar = 1 cm.

Knowledge on the digestive tract of this polyphagous snail is essentially morphological and several specializations have been found [4] (Figure 1): (a) the buccal cavity receives the openings of a pair of salivary glands, (b) the esophagus has a pair of ventro-lateral pouches and an expanded crop in its medial portion that retains food during digestion, (c) a three-chambered stomach, which comprises a muscular gizzard, a vestibule that receives the openings of the midgut gland and the style sac, (d) a thin gut, (e) a coiled gut, and (f) the rectum with an anal gland.

The epithelial cells of the midgut gland of *P. canaliculata* host two types of endosymbiotic pigmented corpuscles which are considered morphotypes of the same organism and are identified as C and K corpuscles [5], [6], [7], [8]. The possible role of this endosymbiont in protein digestion was suggested by the unexpected finding of protease activity in extracts of C corpuscles isolated from the midgut gland of this snail (Vega, unpublished).

Proteolytic enzymes have been studied in vetigastropods (genera *Haliotis* and *Megathura* [9], [10]), stylommatophoran pulmonates (genera *Helix*, *Elona*, *Archachatina, Arion and Dermoceras* [11], [12], [13], [14]), opistobranchs (genera *Hermissenda* and *Aeolidia*, [15]) and in an architaenioglossan (genus *Viviparus* [16]), but lack of proteases has been reported in the ampullariids *Pila virens* [17] and *Pomacea canaliculata* [4], the species which is studied here.

Hydrocarbon hydrolytic enzymes (endo-1,4-β-D-xylanase, α and β -mannosidase, β -N-acetylglucosaminidase, β -galactosidase and α-fucosidase) have been purified from whole animal extracts of *P. canaliculata* [18], [19], [20], [21]. Also, two cellulase cDNAs (GHF10-Pc1 and GHF10-Pc3) belonging to glycoside hydrolase family 10 (GHF10) were isolated and characterized from stomach tissue [22] but, to our knowledge, there is no available information about other enzymes that can participate in food digestion in this snail.

Here we studied (a) the occurrence, diversity and origin of host and endosymbiotic proteases in the digestive tract of *P. canaliculata*, (b) their sensitivity to different protease inhibitors, (c) their optimal pH and temperature ranges of activity, and (d) their cellular distribution in the midgut gland using *in situ* zymography.

## Materials and Methods

### Animals and Culture Conditions

Adult snails (shell length 35–40 mm) from a cultured strain of *P. canaliculata* were used [8]. Room temperature was regulated (23–25°C) and artificial lighting was provided 14 h per day. The animals were maintained in aquaria containing 2 L of tap water and the aquarium water was changed thrice weekly. Unless otherwise indicated, animals were fed *ad libitum* with lettuce from Monday through Friday and this was supplemented with high protein fish food pellets (40% total protein content; Peishe Car Shulet®, Argentina) on Thursday and with excess toilet paper on Friday.

### Luminal Protease Activity

#### Snail acclimation

Animals were acclimated to feed exclusively on fish food pellets for 48 h, after which they were fasted for 24 h. After fasting, each animal was isolated in a vessel containing 70 mL water and 3 food pellets; 90 min after the first pellet was swallowed, each animal was immersed in an ice bath during 10 min to minimize pain and then the shell was cracked and the samples were obtained. The fish food pellets were approximately cubical (∼2.5 mm wide) and each one could be swallowed at once by the snails, without any visible fragmentation or spilling. The snails consumed all the offered food pellets during the 90 min period preceding ice-bathing.

#### Sampling

Immediately, after shell removal, an autostatic forceps was fixed on the posterior esophagus to prevent any passage of contents between the crop and the stomach during sampling. The crop and the style sac contents were collected with a 1 mL syringe by puncturing the walls with an 18-gauge needle. The coiled gut content was collected by gentle squeezing the sectioned gut.

For protein extraction, samples from the crop, style sac and coiled gut contents and of 3 snails were thoroughly dispersed in 750 µl of an extraction buffer (100 mM Tris-HCl, 7.5 mM NaCl, 0.25% Triton X-100, pH 7.4), centrifuged at 10000 *g* for 15 min at 4°C, and the supernatants were kept at −80°C until use.

#### Protease activity

Protease activity was determined in the thawed extracts (N = 10) by spectrophotometry of the colored components generated by digestion of azocasein [23]. Hundred µL of protein extracts (crop content = 10.5–60 mg protein/mL; style sac content = 10.9–56.6 mg protein/mL; coiled gut content = 4.6–11 mg protein/mL) were mixed with 100 µL of a reaction buffer (0.5 M Tris-HCl, 2 M NaCl and 0.05 M CaCl_2_, pH 7.4), 500 µL of 2.5% azocasein as substrate and completed with sterile bidistilled water to final volume of 1 mL. The incubation conditions were 25°C, pH 7.4 and 24 h, unless otherwise indicated. A mix of reagents without sample was used for blanks. The reaction was stopped with 500 µL of 5% trichloroacetic acid and centrifuged at 10000 *g* for 10 min. The supernatant obtained was diluted (1∶1; v/v) in 2% NaOH and read at 440 nm in a Helios® Gamma spectrophotometer. Protein concentration in samples was determined according to the Lowry method [24] and specific protease activity was expressed as international milliunits per milligram of protein (mIU/mg protein) using the Lambert-Beer equation and the azocasein E^1%^ coefficient supplied by the manufacturer.

Also, azocasein fragments resulting from digestion (1 mg protein per sample) were revealed by polyacrylamide gel (15%) electrophoresis using Mini Protean II gel system (Bio-Rad Laboratories, Inc). Electrophoresis was performed according to Sambrook et al. [25] but the samples were processed under cold and non-reducing conditions (i.e., not treated with β-mercaptoethanol or dithiothreitol). After protein digestion, 5 µL of reaction mixture (containing 5 µg of luminal content and 62.5 µg azocasein substrate) were diluted in loading buffer (50 mM Tris-HCl, 2% SDS, 0.1% bromophenol blue and 10% glycerol, pH 6.8). Also, azocasein substrate (62.5 µg) and extracts of luminal contents (5 µg) were loaded alone and used as controls.

Each gel was run at 30 mA and was then stained with 0.75% Coomassie Brilliant Blue R-250 in a water-methanol-acetic acid solution (45∶45∶10).

Also, exopeptidase activity was estimated by measuring the increase in free aminoacid concentration of the samples after digestion of bovine serum albumin (BSA, Sigma, A4503) with extracts of crop, style sac and coiled gut contents (N = 5). Aminoacids were measured by the ninhydrin reaction [26], [27]. For such purpose, 200 µL of each extract were mixed with 10 µL of 2% BSA and were incubated at 35°C for 20 min. The reaction was stopped with 1 mL of 1.2 M trichloroacetic acid (TCA) and the mixture was centrifuged at 10000 *g* for 10 min. Afterwards, 200 µL of the supernatant were mixed with 100 µL of 0.2 mM KCN in 2M acetate buffer (pH = 5.4) and 100 µL of 1.75% ninhydrin (Sigma, N4876) in 100% ethanol. Then the mixture was boiled for 10 min and chilled on ice for 1 min before the addition of 500 µL of 50% ethanol. Measurements were made against a standard curve of L-leucine. Absorbance was read at 570 nm in a Helios Gamma spectrophotometer. Exopeptidase activity was expressed as µmols of aminoacids released/min incubation/mg protein (mean ± SEM).

### Diversity and Tissue Origin Of Luminal Proteases

Acclimation and sacrifice of the animals, and the procedures for sampling the content of the crop, style sac and coiled gut were as described above. Also, both salivary glands and ∼100 mg of the midgut gland and coiled gut tissue were obtained. For protein extraction, tissues were pooled (22 pools, 3 animals each) and homogenized in 750 µl of the protein extraction buffer in an Ultraturrax® homogenizer. These extracts were used for zymographic determinations of apparent molecular weights.

Zymography was made in 10% polyacrylamide gels that were copolymerized with gelatin (Sigma G7765; 1 mg/mL) and 75 µg of the extracted protein were loaded per lane and electrophoresis was run at 4°C under non-reducing conditions. The gelatinolytic activity was revealed according to Sang *et al.* [28] with minor modifications. Briefly, the gels were rinsed for 15 min in a 2.5% Triton X-100 solution followed by three washes in distilled water. Then the gels were pre-incubated in reaction buffer for 30 min, after which the buffer was changed and the gel was incubated overnight in a metabolic shaker (45 rpm at 25°C). Gelatinolysis was revealed by Coomassie Brilliant Blue staining so that protease activity appeared as white areas over a blue background. The apparent molecular weight of proteases was estimated using a linear curve between the relative mobility and the log of different molecular weight markers (20, 29, 45, 66, 97, 116 and 205 kDa; Sigma T9767, C2273, A7642, A7517, P4649, G8511, M3889 respectively). Apparent molecular weights were expressed as mean ± SD.

### Protease Activity in Endosymbiotic Corpuscles

Since a 30 kDa protease was found in extracts of the midgut gland and the style sac (that receives the outflow of the midgut gland containing the endosymbiotic corpuscles) the hypothesis of an endosymbiotic origin of this protease was tested. For such purpose, five pools (2 acclimated snails each) of isolated C and K corpuscles, midgut gland tissue and style sac content were made. Samples from tissues and contents were obtained as already described, while the endosymbiotic corpuscles were isolated from ∼1 g samples of the midgut gland as previously reported [8]; the purity of fractions was microscopically controlled and only those C fractions containing less than 5% of K corpuscles, as well as those K fractions containing less than 1% of C corpuscles were used. The tissue and corpuscular samples were thoroughly crushed in a porcelain mortar using liquid nitrogen and then dispersed in 750 µl of protein extraction buffer, centrifuged, and supernatant samples were processed for gel zymography.

### *In situ* Zymography

Also, midgut gland samples for *in situ* protease zymography were obtained from acclimated snails. They were fixed in Beckstead’s solution [29] and dehydrated through an ethanol series, embedded in paraffin and 5 µm sections were obtained and processed for *in situ* zymography [30] using DQ-gelatin as substrate (D12054, Molecular Probes, Invitrogen). Tissue sections were deparaffinized in xylene, rehydrated in ethanol 96° and bidistilled water, and placed in reaction buffer (see protease activity) for 10 min at room temperature. Then, each tissue section was preincubated in reaction buffer for 1 h at room temperature. Fifty microliters of a 20 µg/mL DQ-gelatin solution was then added to each slide and they were incubated for 2 h at 35°C in a humid chamber. The remaining substrate was then rinsed with bidistilled water and the sections was post-fixed in neutral buffered formalin (4% formaldehyde, 0.33 mM NaH_2_PO_4_, 0.45 mM Na_2_HPO_4_, pH = 7) for 20 min. Finally, each section was rinsed for 5 min in phosphate buffer saline (PBS, 3.4 mM NaCl, 0.067 mM KCl, 0.25 mM Na_2_HPO_4_, 0.046 mM NaH_2_PO_4_, pH = 7) and mounted in glycerol-PBS (90∶10; v/v) containing 5 mg/mL propyl-gallate (P3130, Sigma) [31]. Similarly treated sections, but that were not exposed to the substrate were used as controls. The mounted sections were kept in darkness until epifluorescence was observed and photographed in Nikon Eclipse 80i Microscope using Nikon DS-Fi1-U3 camera and Nikon NIS-ELEMENT Imagen Software for image acquisition.

### Effects of Different Inhibitors on Luminal Protease Activity

The effect of different inhibitors on protease activity was studied in contents of different parts of the digestive tract (crop, style sac, coiled gut). Nine pools of extracts were obtained as already described for each studied portion of the digestive tract. Hundred microliters of each pool (crop content = 10.5–60 mg/mL; style sac content = 10.9–56.6 mg/mL; coiled gut content = 4.6–11 mg/mL) were mixed with the following inhibitors dissolved in reaction buffer at the following final concentrations: 800 nM aprotinin (serine-protease inhibitor, Sigma A1153), 10 µM E64 (cysteine-protease inhibitor, Sigma E3132), 1.45 µM pepstatin A (aspartic-protease inhibitor, Sigma P5318) and 1 mM EDTA (metalloprotease inhibitor, Sigma ED2SS) for 2 h. Extracts without any inhibitor were used as control. Residual protease activity of each experimental group was expressed as % of the protease activity observed in the control group (100%).

Also, extracts of gut contents were obtained and the inhibition produced by each inhibitor (same concentrations above) was evaluated by gel zymography on the different protease bands.

### Effect of the pH and Temperature on Luminal Protease Activity

Extracts of crop, style sac and coiled gut contents were used. Specific protease activity was determined at different pH values (10 pools, 3 acclimated snails each) and temperatures (7 pools, 3 acclimated snails each). Optimal pH range was determined by incubating the sample at 25 °C in the reaction buffer previously adjusted to pH values 5.5, 6.6, 7.5, 8.5, 9.5 or 10.5. The optimal temperature range was determined by incubating the sample at temperatures ranging from 18 to 40°C, at pH 7.4 for 24 h. Protease activity was determined and expressed as percentage of the maximal observed activity.

For determining luminal pH values, samples of the luminal contents of crop, style sac and coiled gut from two acclimated animals were pooled, diluted in ASMT type I water (1/25, v/v) and pH was measured immediately at 24°C with a Beckman Φ300 pHmeter. Ten pools were used for determinations and mean pH values (± SEM) were calculated for each digestive tract portion.

### Tissue L-ala-N-aminopeptidase Activity in Portions of the Digestive Tract

Five pools of crop, style sac and coiled gut tissues, cleaned of its contents in HEPES-mannitol buffer (1 mM HEPES-KOH, 150 mM mannitol, pH 7.6) were used to determine specific aminopeptidase activity according to Ciminari *et al.* [32]. Thawed samples (20 µL) of each tissue extract were mixed with 1 mL of HEPES-mannitol buffer containing 2 mM L-alanine-p-nitroanilide hydrochloride (Sigma, A9325) and incubated at 25°C for 20 min and then the reaction was stopped with 3 mL acetic acid. The standard curve was made using different dilutions of 4-nitroaniline (Sigma, N2128). The absorbance of 4-nitroalinine released was read at 384 nm in a Helios Gamma spectrophotometer. Specific activity was expressed as international milliunits per milligram of protein. Results were expressed as mean ± SEM.

### Statistical Analyses

**Figure 2:**
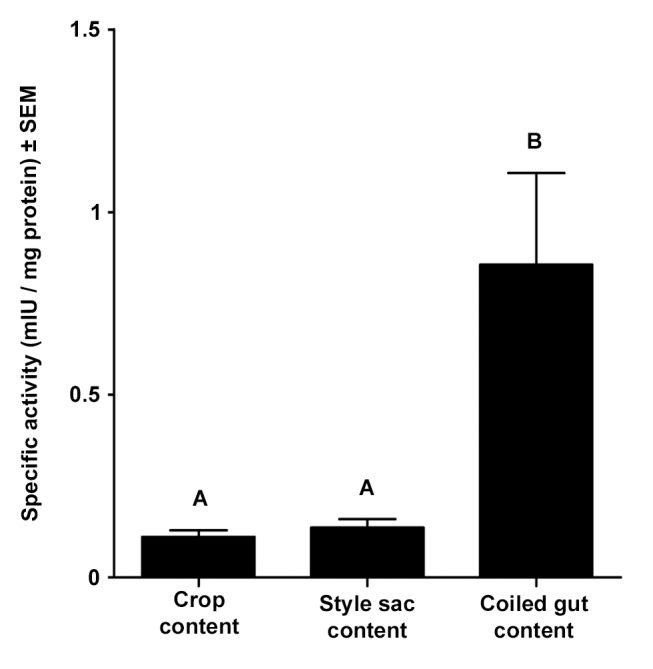
Specific protease activity in extracts of digestive tract contents. Different letters indicate statistically significant differences between groups (mean ± SEM, N = 10, Kruskal –Wallis test followed by Dunn’s test, p<0.05).

For multigroup comparisons, the distribution of variables was first evaluated by Kolmogorov–Smirnov’s normality test, and equal variance Bartlett’s test was used to evaluate homogeneity of variances for each set of experimental variables before applying one-way ANOVA and the Tukey test as a post-hoc analysis. Multigroup comparisons for specific protease activity (Figure 2) were made by Kruskal–Wallis test and Dunn’s test as post hoc analysis, since the experimental groups presented unequal variances. Confidence intervals (99%) were used to assess the significance of differences from the control group without inhibitors (100%). All data analyses were performed using InfoStat®, version 2009. Significance level was fixed at p<0.05.

## Results

### Luminal Protease Activity

**Figure 3:**
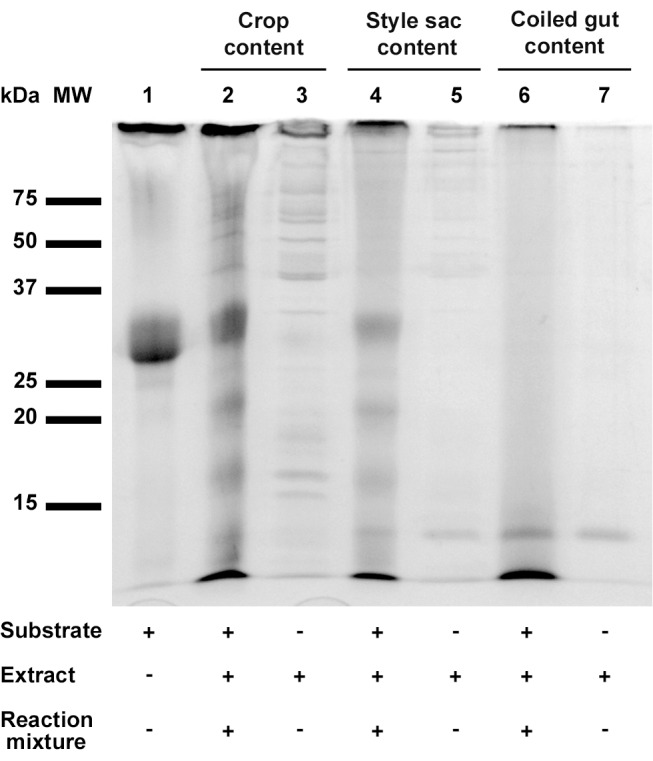
Azocasein digestion by extracts of crop, style sac and coiled gut contents. SDS-15% polyacrylamide gel electrophoresis showing the digestion products of azocasein. MW: molecular weight markers. Azocasein substrate (62.5 µg) was loaded in lane 1. Extracts of the different regions of the digestive tract (5 µg proteins) were loaded in lanes 3, 5 and 7. The reaction mixture initially containing 62.5 µg of azocasein and 5 µg of protein in each extract were loaded in lanes 2, 4 and 6.

Protease specific activity was detected in luminal contents from all sampled regions of the digestive tract. Mean specific activity in the coiled gut was about 6-times higher than in the crop and the style sac, and the differences were statistically significant (Figure 2, Kruskal-Wallis test). The fragmentation pattern of the azocasein substrate by luminal protease activity varied in the different digestive tract regions (Figure 3). The azocasein substrate was cut into four smaller fragments by the crop and style sac extracts indicating endopeptidase activity (Figure 3, lanes 2 and 4), while the substrate was completely digested by the coiled gut content (Figure 3, lane 6). However, this difference was not a consequence of the high activity present in this gut region, since a similar fragmentation pattern was observed when the assay was normalized by using the same specific activity to each lane (Figure S1).

Exopeptidase activity was detected in the crop, style sac and coiled gut content, but it was much higher in the latter (3.5±1.3; 4.6±1.1; 218.6±46.7, respectively). Differences were significant between the coiled gut content and those of the other studied organs (one-way ANOVA, Tukey test).

### Diversity and Tissue Origin of Luminal Proteases

**Figure 4:**
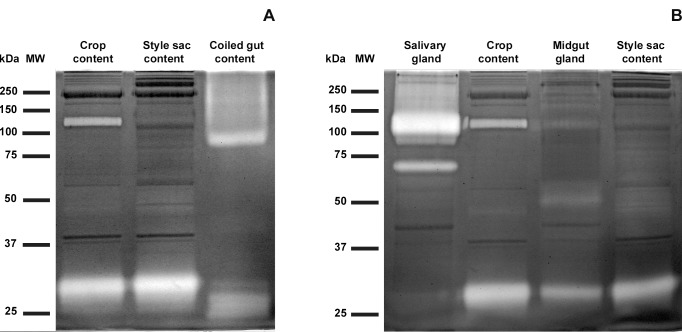
Diversity and tissue origin of digestive tract proteases. Zymographies (10% polyacrylamide gels copolymerized with 1 mg/mL gelatin) showing protease activity (clear bands where gelatin was digested) and proteins with no protease activity (dark bands); Coomassie Brilliant Blue staining. (**A**) High and low molecular weight bands showing protease activity in digestive tract contents. (**B**) Correlative protease activity in salivary gland tissue and crop content, and in midgut gland tissue and style sac content. MW = molecular weight markers. Each lane was loaded with 75 µg of protein.

The diversity and frequency of luminal protease bands is shown in Figure 4A and Table 1, respectively. A 30 kDa protease band was the most frequently found in the lumen of the digestive tract. A 125 kDa protease band was also present in extracts of the crop and style sac content in most snails but was always absent in the coiled gut. Two high molecular weight proteases (145 and 198 kDa) were only but frequently found in the coiled gut content.

**Table 1:** Occurrence of proteases in the digestive tract (% of cases, N = 22).

Proteases (kDa)	Crop content	Style sac content	Coiled gut content	Midgut glandtissue	Salivary gland tissue	Coiled gut tissue
**28±1**	0	9	100	9	0	0
**29±1**	0	0	0	0	0	95
**30±2**	100	100	100	72	0	0
**70±2**	0	0	0	0	77	0
**125±5**	82	41	0	0	100	0
**145±5**	0	0	86	0	0	0
**198±5**	0	0	64	0	0	0

A 28 kDa protease band was found less frequently than the 30 kDa protease, but always together with it, and also, a weak protease activity was sometimes found between 40–55 kDa (Figure S2).

Protease activity in tissue extracts (Figure 4B) suggests that the salivary gland is the origin of the 125 kDa protease band, while the midgut gland is the origin of the ubiquitous 30 kDa protease band and of the less frequent 28 and 40–55 kDa ones. Salivary gland and coiled gut extracts showed frequently proteases of 70 kDa (Figure 4B) and 29 kDa (Figure S2), respectively, but it did not appear in the intestinal lumen.

The high molecular weight proteases found in the coiled gut lumen were not found in any of the extracts from salivary gland, midgut gland and coiled gut.

### Endosymbiotic Protease Activity

**Figure 5:**
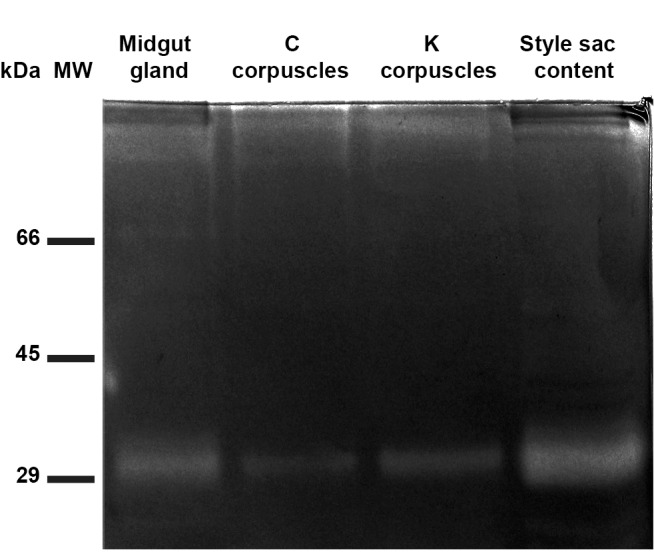
Endosymbiotic origin of the 30 kDa protease. Zymography (10% polyacrylamide gel copolymerized with 1 mg/mL gelatin) of extracts of midgut gland, C and K corpuscles, and style sac content. MW = molecular weight markers. Each lane was loaded with 75 µg of protein.

Zymograms of extracts from midgut gland, C and K corpuscles and the style sac content (Figure 5) showed a protease band with an apparent molecular weight of 30 kDa (five independent replicates were run). Also, a 45 kDa protease band appeared in 2 of these cases in the style sac content, as well as two small and unusual ∼21 and ∼23 kDa protease bands (not shown in Figure 5).

### Inhibition of Luminal Proteases

Results are shown in Table 2. Only the serine-protease inhibitor aprotinin caused a significant effect on total protease activity in the crop and style sac contents (99% confidence intervals). Also, both aprotinin and EDTA produced some inhibition of total protease activity in the coiled gut, but the difference was not statistically significant.

**Table 2:** Residual protease activity under different inhibitors.

Intestinal contents	No inhibitor (control)	Aprotinin (serine-protease inhibitor)	E64 (cysteine-protease inhibitor)	Pepstatin (aspartic-protease inhibitor)	EDTA (metalloprotease inhibitor)
**Crop**	100	45±4*	125±18	123±7	117±3*
**Style sac**	100	38±3*	99±5	108±5	106±4
**Coiled gut**	100	84±9	100±8	98±7	78±6

Percent inhibition (mean ± SEM) was calculated as residual protease activity in each group exposed to an inhibitor, divided by protease activity in the control group (without inhibitor) and multiplied by 100. N was 9 in each group. Ninety nine % confidence intervals were used to assess the significance of differences (asterisks) from the control group.

**Figure 6:**
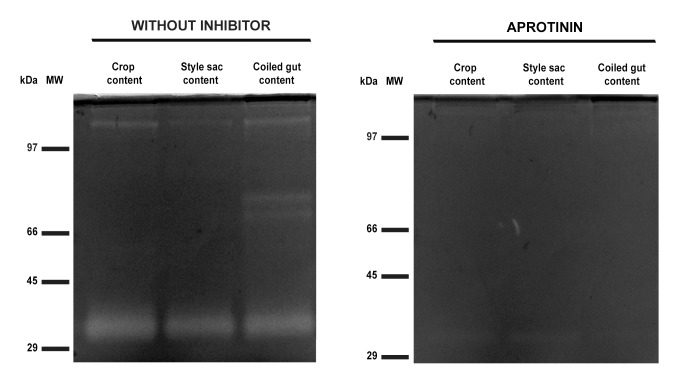
Inhibition of serine-proteases. Zymographies (10% polyacrylamide gels copolymerized with 1 mg/mL gelatin) of contents of the crop, style sac and coiled gut that were incubated without or with aprotinin (800 nM). MW: molecular weight markers. Each lane was loaded with 75 µg of protein.

The inhibitory effect of aprotinin on luminal digestive tract proteases was also shown in zymograms, since inhibition of each particular protease band (30, 125, 145 and 198 kDa) occurred in all studied portions of the digestive tract (Figure 6). However, no inhibition was observed in zymograms in presence of E64, pepstatin A or EDTA (Figure S3).

### Effect of the pH and Temperature on Luminal Protease Activity

**Figure 7:**
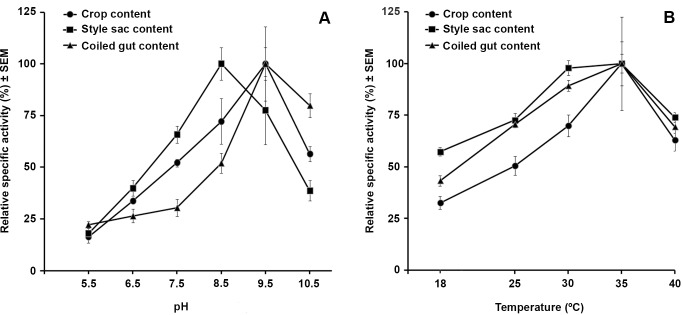
Optimal pH and temperature of protease activity in digestive tract contents. Specific protease activity of digestive tract contents at different pH (**A**) and temperature (**B**) values. Results were expressed as mean ± SEM. N was 10 for pH determinations and 7 for temperature determinations.

Total protease activity in the three studied portions of the digestive tract was maximal at alkaline pH values (8.5–9.5; Figure 7A) and at temperatures between 30°–35°C (Figure 7B).

The pH values found in the luminal contents were: (a) crop, 5.96±0.09, (b) style sac, 6.17±0.09, and (c) coiled gut, 7.42±0.07) (mean ± SEM, N = 11).

### Tissue L-ala-N-aminopeptidase Activity in Portions of the Digestive Tract

**Figure 8:**
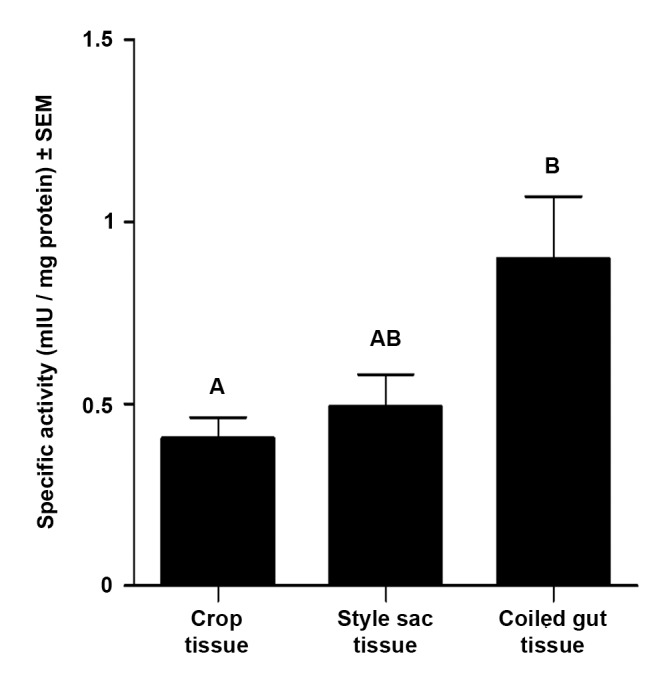
Specific L-alanine-N-aminopeptidase activity in wall tissue of the crop, style sac, and coiled gut. Different letters indicate statistically significant differences between groups (mean ± SEM, N = 5, one way ANOVA followed by Tukey test, p<0.05).

Aminopeptidase specific activity in the wall tissue of the crop, style sac, and coiled gut is shown in Figure 8. Statistically significant differences were found between the crop and the coiled gut only.

### *In situ* Protease Activity in the Endosymbiosis and the Midgut Gland

**Figure 9:**
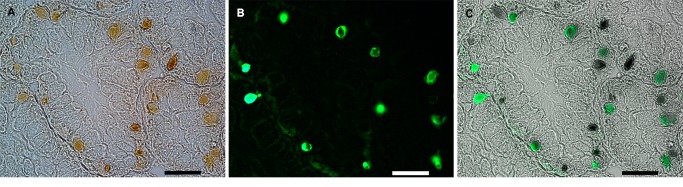
*In situ* zymography of midgut gland. **A**. Light microscopy of several tubulo-acini, showing numerous pigmented endosymbiotic corpuscles. **B**. Green fluorescence (FITC) indicates substrate degradation (DQ-gelatin). **C**. Merging of A and B showing substrate degradation by most endosymbiotic corpuscles. Corpuscles which do not show fluorescence may be either dead symbionts or, more likely, they may be the result of differences in the penetration of reagents. Scale bars = 50 µm.

Fluorescence of degraded substrate (DQ-gelatin) is shown in sections of midgut gland tissue, indicating protease activity. A strong fluorescence signal was emitted by most endosymbiotic corpuscles (Figure 9) but only a very low signal was emitted by the surrounding cytoplasm of host cells. Sections which were not exposed to DQ-gelatin showed similarly low autofluorescence by the cytoplasm of host cells, but endosymbiotic corpuscles emitted no autofluorescence (not shown in Figure 9).

## Discussion

This is the first report of proteases in the digestive tract of ampullariid snails, since earlier authors had failed to detect any protease activity in the gut of ampullariids *P. canaliculata* and *Pila virens* [4], [17]. However, the methods used to detect protease activity were not stated in those early publications.

A 30 kDa serine protease (EC 3.4.21.) from endosymbiotic origin is the most frequently found in the lumen of the digestive tract in *P. canaliculata*. Also, another serine protease (125 kDa) originated in the salivary glands of the host is found in the lumen of the crop and style sac. Furthermore, two high molecular weight serine proteases (145 and 198 kDa) of uncertain origin are found in the coiled gut lumen. And finally, L-ala-N-aminopeptidase activity (EC 3.4.11) is found in the wall tissue of the crop, style sac and coiled gut.

### Salivary Glands

A pair of salivary glands is present in most gastropods and their ducts open into the buccal cavity and are thought to be involved in lubrication and agglutination of food [33]. Also, carbohydrases, proteases, lipases and phosphatases have been found in the saliva of herbivore and omnivore gastropods [10], [34], [35].

Two proteases (70 and 125 kDa of apparent molecular weight) are found in the salivary glands of *P. canaliculata* but only the larger one is found in the crop lumen. The possibility that this protease were synthetized as a 70 kDa subunit and that the secreted 125 kDa protease were a dimer should await further investigation.

### Midgut Gland and Endosymbiotic Corpuscles

The 30 kDa protease is ubiquitously found in the digestive tract of *P. canaliculata*. It is present in extracts of both the midgut gland and the isolated endosymbionts, and activity (as determined by *in situ* zymography) is found in the latter. The endosymbionts are released through the midgut gland ducts into the stomach vestibule, and so a 30 kDa protease activity is found downstream in the style sac and the coiled gut lumen. However, it is also found upstream in the crop content, which is explained by the lack of a sphincter separating the stomach from the posterior esophagus and, probably, by backward flux driven by pumping action of the muscular gizzard. The generation of four azocasein fragments by extracts of the crop and style sac contents suggests that both proteases, endosymbiotic of 30 kDa and salivary of 125 kDa, are indeed endopeptidases.

Also, a 28 kDa protease has been found together with the 30 kDa protease in the coiled gut lumen. It has also been found in two cases in the style sac content and the midgut gland. It is possible that it is a large fragment resulting from partial digestion, but still retaining protease activity. Future research will have to clarify this point. A 45 kDa protease was found in a few cases in both the midgut gland and the style sac content which may be either a short half-life protease or one that is rapidly inactivated during sample processing.

Even though 28–30 kDa proteases have also been reported in other gastropods [36], [37], [38], bivalves [39], [40] and cephalopods [41], [42] none of them has been attributed an endosymbiotic origin.

### Proteases in the Coiled Gut Lumen

Total protease activity in the coiled gut lumen is much higher than in the lumen of the crop and the style sac (Figure 2). The high molecular weight proteases of 145 and 198 kDa are only found in the coiled gut (Figure 3 and Table 1). High exopeptidase activity was also found in the coiled gut.

The origin of the 145 and 198 kDa proteases is not clear, since they were not found either in coiled gut tissue or in extracellular bacteria grown in LB medium from samples of the coiled gut content (data not shown). Some possibilities should await future research: (a) that these proteases are produced and secreted in an inactive form; (b) that particular conditions of the coiled gut environment allow the expression of the 145 and 198 kDa proteases by either the endosymbionts or the extracellular bacteria but that these conditions are not reproduced *in vitro* (LB medium). In this context, the possible role of intestinal bacteria (either probionts or not) in promoting digestion has attracted research in farmed abalones (Vetigastropoda) [43], [44]. However, their quantitative significance as compared with that of host enzymes should await further studies, since a 98–99% reduction of intestinal bacteria by antibiotics was generally unable to effectively reduce proteolytic activity in a pulmonate [45].

In addition the coiled gut tissue presents high L-ala-N-aminopeptidase activity (Figure 8), indicating that it participates in the later stages of protein digestion. Also, a lower activity was found in the crop and style sac tissues. This is the first report of aminopeptidase activity in the digestive tract of an ampullariid snail, even though aminopeptidases are known to occur in the digestive tract of at least three vetigastropods (*Haliotis fulgens*,*H. rufescens,* [37] and *Megathura crenulata* [10], the littorinimorph *Littorina irrorata* [46] and the pulmonate *Biomphalaria straminea* [34].

### Proteases Characterization: Inhibitors and Optimal pH and Temperature

All proteases found in the digestive tract of *P. canaliculata* were significantly inhibited by aprotinin (Table 2 and Figure 6), a broad-spectrum serine protease inhibitor [47]. The mean pH values found in portions of the digestive tract of *P. canaliculata* (crop = 5.96, style sac = 6.17, coiled gut = 7.42) are all below that of circulating hemolymph plasma [48] and would imply the existence of mechanisms for proton secretion into the gut of this snail.

It may be worth mention that similar, predominantly acidic pH values have been reported in the land pulmonates *Helix pomatia* (5.5–6.4), *Helix aspersa*, (6.1–7.4), *Elona quimperiana* (5.3–6.6) [14] and *Arion ater* (5.5–6.0) [11], and in the littorinimorph *Littorina irrorata* (5.8–7.3) [46].

It is intriguing, however, that optimal specific protease activity in this study was found at higher pH values (style sac = 8.5, crop and coiled gut = 9.5; Figure 7A). Also, the optimal temperature values (30 °C for the style sac content and 35°C for the crop and coiled gut contents, Figure7B) were only marginally overlapped with the thermal range of habitats occupied by *Pomacea canaliculata* (10°C to 30°C) [49], [50]. Taken together, these data indicate that the proteases reported here are working effectively on dietary proteins at suboptimal conditions of pH and temperature.

### Conclusions

Protease activity found in the contents of different digestive tract regions indicates that extracellular digestion of dietary proteins is significant in *P. canaliculata*. The sequential occurrence of endopeptidases and exopeptidases in the lumen, together with the occurrence of aminopeptidases in tissues, particularly in the coiled gut, may ensure the proper digestion and absorption of proteins in this polyphagous snail.

The existence and physiological role of the 30 kDa endosymbiotic protease reported herein should also be studied in other ampullariid species in which similar pigmented corpuscles occur [5], [51], [52] and suggest a possible functional role for these symbiotic association, which should be added to the detoxifying role of the endosymbiosis that have been proposed elsewhere [53]. Future studies on proteomics and genomics of the 30 kDa protease could help to understand the phylogenetic relationships among the related endosymbionts in ampullariid snails.

## Supporting Information

Figure S1 **Azocasein digestion by extracts of crop, style sac and coiled gut contents. Extracts were diluted to obtain the same specific activity in all incubations.** SDS-15% polyacrylamide gel electrophoresis showing the digestion products of 1.25% azocasein by 0.1 mIU/mg of each extract.MW: molecular weight markers. Azocasein substrate (15 µL) was loaded in the first lane. Fifteen µL of each reaction mixture were loaded in lanes 2–4.
(TIF)

Figure S2 **Proteases in the tissue and content of coiled gut.** Zymography (10% polyacrylamide gel copolymerized with 1 mg/mL gelatin) showing protease activity in a coiled gut content and tissue obtained after the acclimation of snails. MW = molecular weight markers. Each lane was loaded with 75 µg of protein.
(TIF)

Figure S3 **Zymograms of gut contents in presence of E64, pepstatin A or EDTA.** Zymograms (10% polyacrylamide gels copolymerized with 1 mg/mL gelatin) of contents of the crop, style sac and coiled gut contents that were incubated with or without E64 (10 µM), or pepstatin A (1.45 µM), or EDTA (1 mM). MW: molecular weight markers. Each lane was loaded with 75 µg of protein.
(TIF)
