# Correction: Endosymbiotic and Host Proteases in the Digestive Tract of the Invasive Snail *Pomacea canaliculata*: Diversity, Origin and Characterization

**DOI:** 10.1371/annotation/a38fc487-c159-4bdc-bced-b46a30464dfd

**Published:** 2013-07-08

**Authors:** Martín S. Godoy, Alfredo Castro-Vazquez, Israel A. Vega

The name of the second author was spelled incorrectly. The correct name is: Alfredo Castro-Vazquez. The correct citation is: Godoy MS, Castro-Vazquez A, Vega IA (2013) Endosymbiotic and Host Proteases in the Digestive Tract of the Invasive Snail *Pomacea canaliculata*: Diversity, Origin and Characterization. PLoS ONE 8(6): e66689. doi:10.1371/journal.pone.0066689.

