# Supplementary figures and images for: Endosymbiotic and Host Proteases in the Digestive Tract of the Invasive Snail Pomacea canaliculata: Diversity, Origin and Characterization
Source: PLoS One. 2013 Jun 20;8(6):e66689. doi: 10.1371/journal.pone.0066689 (PMC3688566; doi:10.1371/journal.pone.0066689)

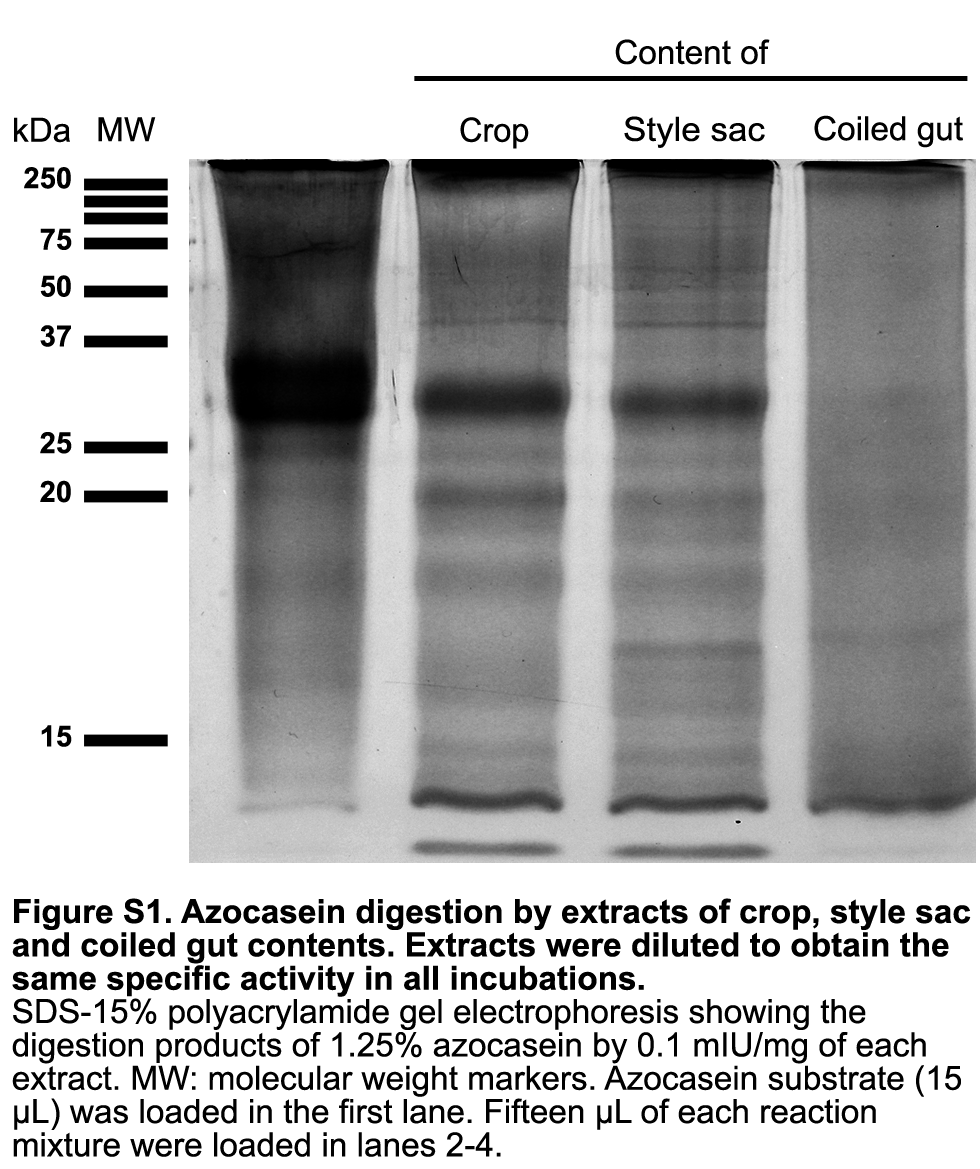

Supplement: Figure S1 — Azocasein digestion by extracts of crop, style sac and coiled gut contents. Extracts were diluted to obtain the same specific activity in all incubations. SDS-15% polyacrylamide gel electrophoresis showing the digestion products of 1.25% azocasein by 0.1 mIU/mg of each extract.MW: molecular weight markers. Azocasein substrate (15 µL) was loaded in the first lane. Fifteen µL of each reaction mixture were loaded in lanes 2–4. (TIF) [file pone.0066689.s001.tif]

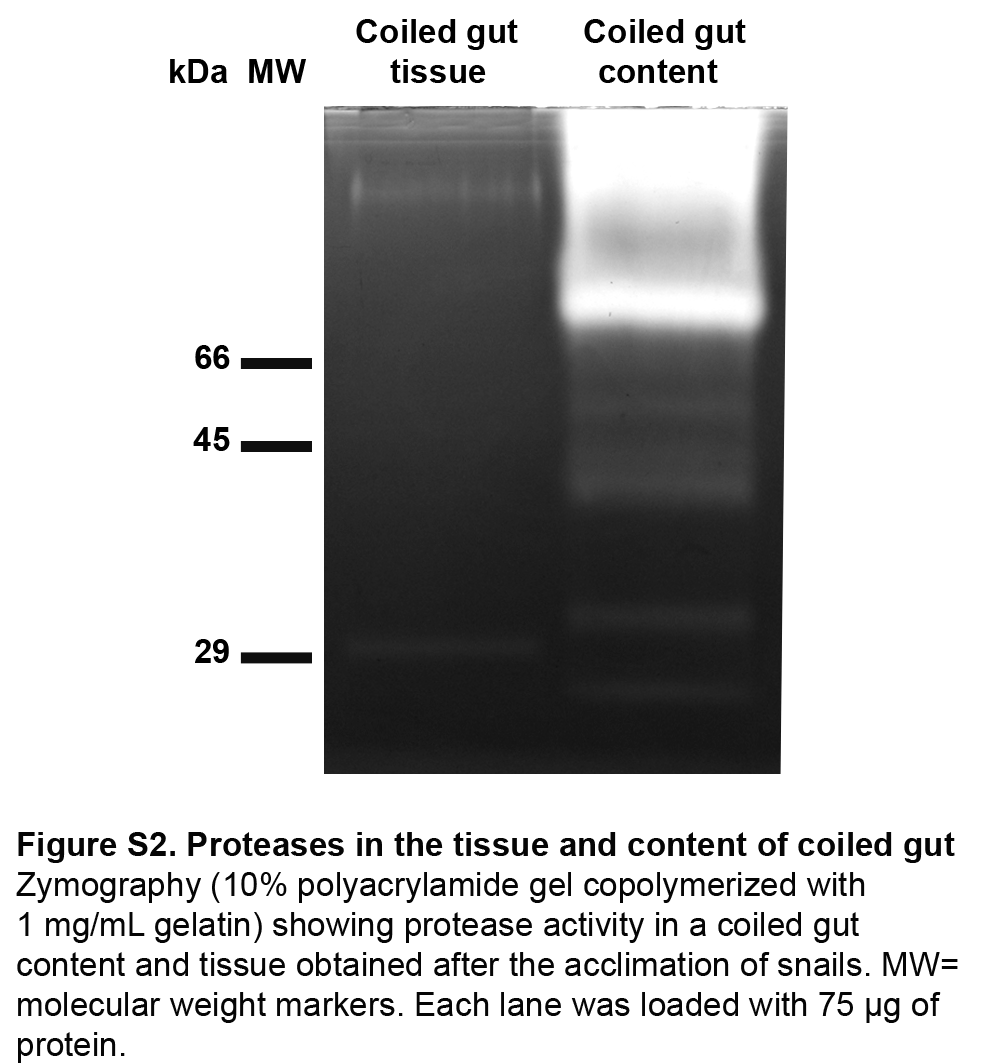

Supplement: Figure S2 — Proteases in the tissue and content of coiled gut. Zymography (10% polyacrylamide gel copolymerized with 1 mg/mL gelatin) showing protease activity in a coiled gut content and tissue obtained after the acclimation of snails. MW = molecular weight markers. Each lane was loaded with 75 µg of protein. (TIF) [file pone.0066689.s002.tif]

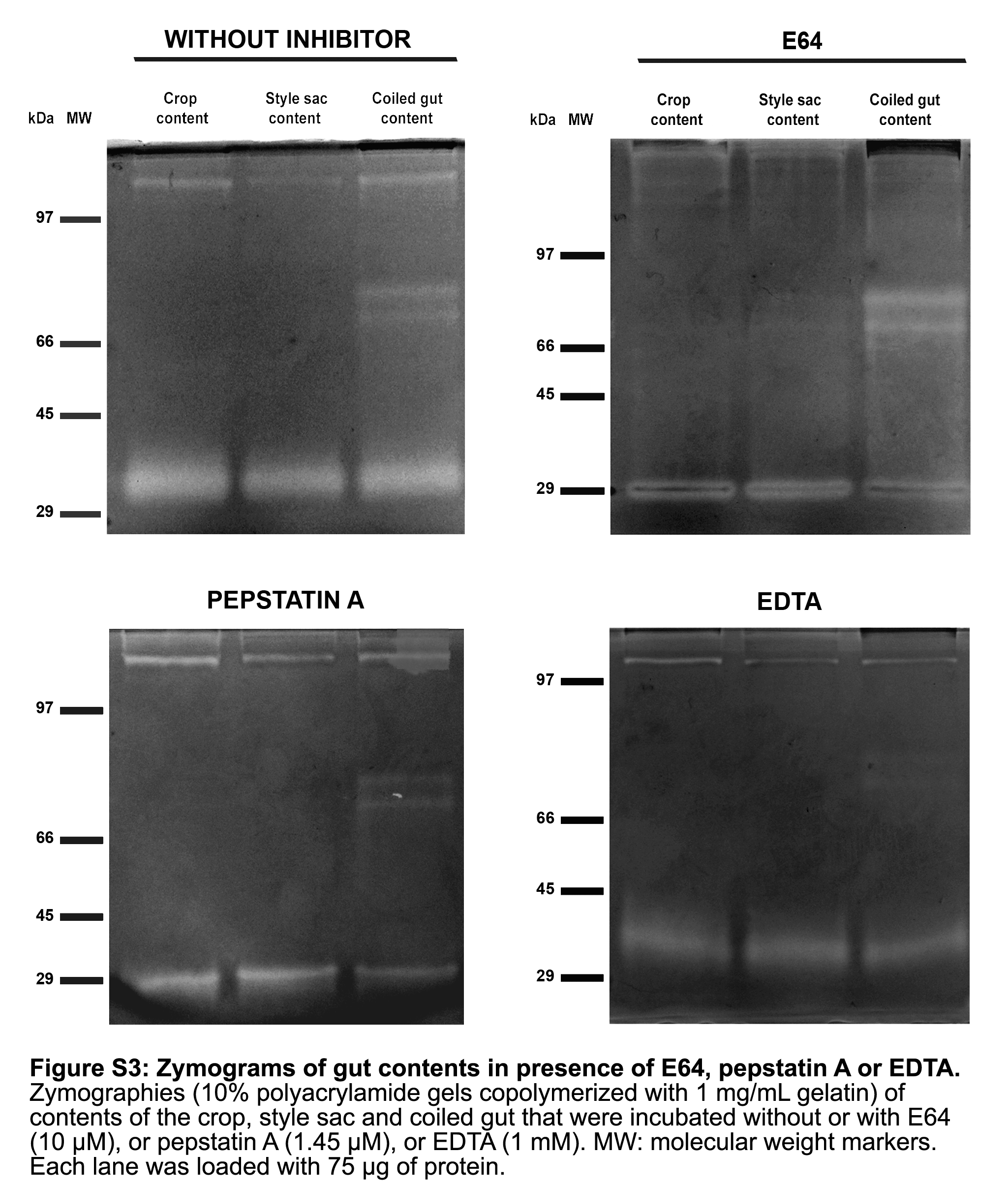

Supplement: Figure S3 — Zymograms of gut contents in presence of E64, pepstatin A or EDTA. Zymograms (10% polyacrylamide gels copolymerized with 1 mg/mL gelatin) of contents of the crop, style sac and coiled gut contents that were incubated with or without E64 (10 µM), or pepstatin A (1.45 µM), or EDTA (1 mM). MW: molecular weight markers. Each lane was loaded with 75 µg of protein. (TIF) [file pone.0066689.s003.tif]
